# Supplementary figures and images for: Quality control implementation for universal characterization of DNA and RNA viruses in clinical respiratory samples using single metagenomic next-generation sequencing workflow
Source: BMC Infect Dis. 2018 Oct 29;18:537. doi: 10.1186/s12879-018-3446-5 (PMC6206636; doi:10.1186/s12879-018-3446-5)

## Slide 1
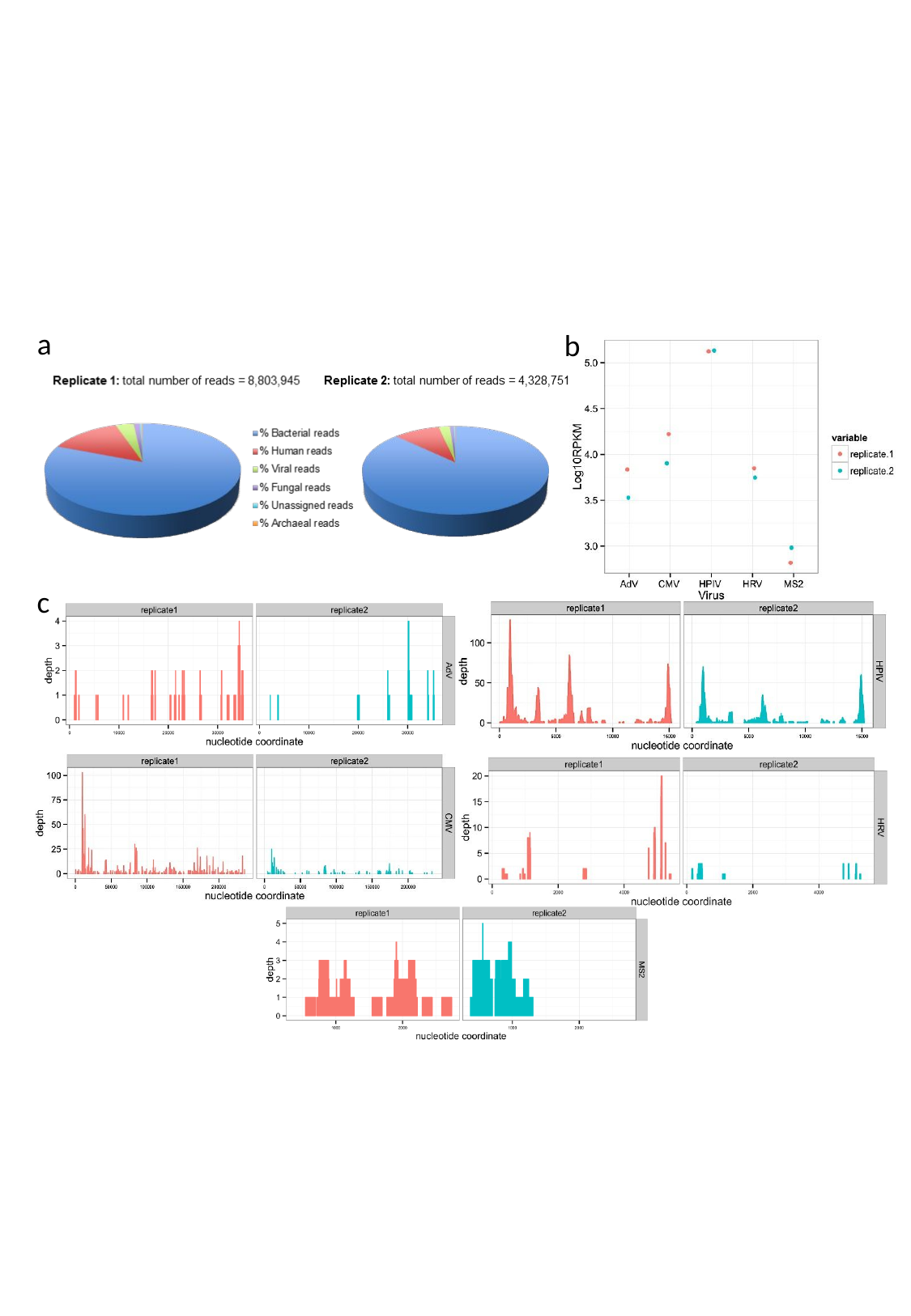

a
b
c

Supplement: Supplementary file 3 — Metagenomic NGS results for duplicates of sample # 25. Sample # 25 corresponds to a clinical respiratory sample tested positive for 2 DNA viruses (adenovirus, cytomegalovirus) and 2 RNA viruses (human parainfluenza virus, human rhinovirus) using real-time PCR. This sample was analyzed twice using our single metagenomic workflow (replicate 1 and replicate 2). a) Pie charts show classification of reads into human, bacteria, viruses, fungi, archea and unknown categories (unassigned reads). b) Normalized read counts (RPKM) for each targeted virus (viruses detected with real-time PCR) and for internal quality control (MS2 bacteriophage). c) Coverage plot of targeted viral genomes and internal quality control (MS2 bacteriophage). Sequencing reads were mapped on ezVIR viral database that identified human adenovirus C-2 (accession number: KF268130.1), cytomegalovirus (accession number: GQ396662.1), human parainfluenza virus 3 (accession number: KF687321.1), human rhinovirus C (accession number: JF317014.1) and MS2 bacteriophage (accession number: NC_001417.2). (PPT 283 kb) [file 12879_2018_3446_MOESM3_ESM.ppt]
